# Electrochemical Sensors Based on Carbon Nanomaterial Used in Diagnosing Metabolic Disease

**DOI:** 10.3389/fchem.2020.00651

**Published:** 2020-08-11

**Authors:** Congcong Zhang, Xin Du

**Affiliations:** Shandong Provincial Key Laboratory of Animal Resistance Biology, Key Laboratory of Food Nutrition and Safety, College of Life Sciences, Shandong Normal University, Jinan, China

**Keywords:** metabolic disease, biomarkers, electrochemical sensors, carbon nanomaterials, detection

## Abstract

Metabolic diseases have become common diseases with the improvement of living standards because of changed dietary habits and living habits, which seriously affect health. Currently, related biomarkers have been widely used as important indicators for clinical diagnosis, treatment, and prognosis of metabolic diseases. Among all detection methods for biomarkers of metabolic diseases, electrochemical sensor technology has the advantages of simplicity, real-time analysis, and low cost. Carbon nanomaterials were preeminent materials for fabricating electrochemical sensors in order to enhance the performance. In this paper, we summarize the research progress in the past 3 years of electrochemical sensors based on carbon nanomaterials in detecting markers of metabolic diseases, which include carbon nanotubes, graphene, carbon quantum dots, fullerene, and carbon nitride. Additionally, we discuss the future prospects for this field.

## Introduction

Human life activities are inseparable from metabolism, and the stability of metabolic activity plays a very important role in maintaining human health. Once metabolic dysfunction occurs, it can lead to the occurrence of a variety of diseases. Metabolic diseases are usually caused by metabolic problems, including metabolic disorders and metabolic exuberance (Cani, 2019; Sanna et al., 2019). Metabolic diseases mainly include diabetes mellitus (DM) (Maiese, 2020), non-alcoholic fatty liver disease (NAFLD) (Colca, 2020), gout (Oh et al., 2019), vitamin deficiency (Kim et al., 2015), diabetic ketoacidosis (Musso et al., 2019), and other diseases which can also lead to a variety of complications. The metabolic disease will further cause atherosclerosis and other cardiovascular diseases if the patient is not treated in time. These diseases not only affect normal diet and activities but also pose a threat to health and normal life (Clemmensen et al., 2019).

Taking DM as an example, DM is a group of metabolic diseases characterized by hyperglycemia (Rosik et al., 2020). The traditional diagnostic methods for detecting infectious diseases include routine blood test, biochemical test, routine urine test, and glucose tolerance tests that are often expensive, time-consuming, and labor-intensive (Sahin et al., 2018; Salek-Maghsoudi et al., 2018). Compared with traditional detection methods, electrochemical biosensor technology has been increasingly applied due to its unique advantages (Du and Zhou, 2018; Maduraiveeran et al., 2018). Nanostructured carbon-based materials, such as carbon nanotubes (Chen et al., 2016a,b), graphene (Janegitz et al., 2017; Abellan-Llobregat et al., 2018; Taniselass et al., 2019), carbon quantum dots (Ren, 2015; Ding et al., 2020), fullerenes (C60) (Kawase et al., 2003), and carbon nitride (Zhang P. et al., 2019) have gained enormous attention in preparing electrochemical biosensors for their high durability, excellent electrical conductivity, and excellent biocompatibility. According to the research progress, biosensors can match or even surpass traditional methods due to the excellent performance of electrochemical sensors (Rombout et al., 2014; Du et al., 2020). The development of nanomaterials and electronic technology has accelerated the development of electrochemical biosensing technique which provides a new method for rapid detecting in biomarkers of metabolic diseases.

Metabolic disease biomarkers, as important indicators for clinical diagnosis, treatment reference, and prognosis judgment, have been widely used (Liang et al., 2010; Liu M. et al., 2018; Wang et al., 2018). For example, ascorbic acid (AA) (Hsine et al., 2020), uric acid (UA) (Iranmanesh et al., 2020), leptin (Guo et al., 2015), 3-hydroxybutyrate (3-HB), and glucose are important biological molecules indispensable to the human body. For instance, the main cause of scurvy is lack of AA. Vitamin D can regulate calcium and phosphorus homeostasis and control bone metabolism, whose deficiency is associated with rickets in children and osteomalacia in adults (Pittas et al., 2019). The deficiency of vitamin D has also been linked to DM (Sassi et al., 2018; Bouillon et al., 2019). Methylglyoxal (MG) can be treated as an emerging biomarker for DM diagnosis; meanwhile, it also plays a crucial role in biological processes in hypertension and nephropathy (Maessen et al., 2015). The concentration of 3-HB is related to diabetic ketoacidosis (Liu et al., 2015; Rodriguez-Gallego et al., 2015). High levels of UA in the blood is the cause of gout (Cui et al., 2011; Ding et al., 2018). Leptin and acetaminophen metabolism can be used as biomarkers for the diagnose of NAFLD (A-Kader et al., 2010; Perez-Perez et al., 2017). Therefore, the rapid and accurate determination of the concentrations of these biomarkers in the human body is of great significance for the study of human physiological functions and the diagnosis and prevention of diseases.

## Principle of Electrochemical Sensor

**Figure 1:**
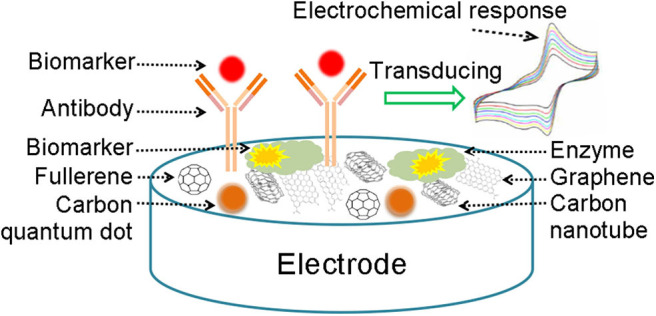
A schematic representation of electrochemical biosensor.

Electrochemical detection method means that the electrochemical biosensor is constructed to realize rapid detection, which is mainly used in biocomponents such as antibodies and enzymes to modify electrodes (Tilmaciu and Morris, 2015). When the original biocomponents react with the specific target analyte, the reaction can be confirmed and measured; meanwhile, electrical signals are generated which are processed by the electronic system and then become the information that we can observe directively. Common detection methods of electrochemical sensor mainly contain linear sweep voltammetry (LSV), cyclic voltammetry (CV), and differential pulse voltammetry (DPV). The principle of electrochemical sensors based on carbon nanomaterials in detecting metabolic disease biomarkers is shown in Figure 1.

## Application of Carbon Nanomaterials in Metabolic Disease Detection

In recent years, increasing attention has been paid to the detection of metabolic disease biomarkers by electrochemical biosensors. Therefore, this paper reviews the latest research progress of several different carbon nanomaterials in electrochemical biosensor detection of common markers of metabolic diseases. The performance of the sensors introduced in this review was summarized as shown in Table 1.

**Table 1:** Comparison of electrochemical sensors based on carbon nanomaterials for detecting biomarkers of metabolic diseases.

**Carbon nanomaterial**	**Disease biomarker**	**Linear range (μM)**	**Detection limit (μM)**	**Reproducibility**	**Stability**	**References**
CNT	UA	0.6–52	0.157	–	–	Rajabi and Noroozifar, 2017
CNT	AA, UA	–	0.65	7%	–	Abellan-Llobregat et al., 2018
CNT	UA	0.2–4, 4–20	0.139	2.20%	95% (2 weeks)	Lv et al., 2019
CNT	AA, UA	200–400, 2–16	40.0, 0.73	0.77%, 2.87%	≥92.5% (15 days)	Zhang S. et al., 2019
CNT	UA	20–2700	2.8	1.30%	90% (24 h)	Fukuda et al., 2020
CNT	UA	7–20	2.4	4.71%	95% (20 days)	Manjunatha et al., 2018
Graphene	UA	0.5–100	0.053	–	91.6% (2 weeks)	Pang et al., 2020
Graphene	UA	0.02–783.6	1.2 × 10^−4^	3.28%	96.42% (30 days)	Sriram et al., 2019
Graphene	UA	50–800	7.32 ± 0.21	4.60%	90.0% (6 months)	Verma et al., 2019
Graphene	AA, UA	0.1–1 × 10^4^	0.046, 0.013	3.2%, 2%	99.4%, 98.4% (15 days)	Hsine et al., 2020
Graphene	AA, UA	1 × 10^3^ to 1.5 × 10^4^	0.049, 0.047	4.2%, 2.7%	–	Demirkan et al., 2020
Graphene	Leptin	1.5 × 10^−7^ to 2.5 × 10^−3^	3.6 × 10^−8^	6.27%	92.32% (7 days)	Cai et al., 2019
Graphene	Acetaminophen	0.05–0.8	1.4 × 10^−4^ (±5 × 10^−5^)	<4.4%	93.11 ± 2% (12 weeks)	Kumar et al., 2019
Graphene	Glucose	1–100	0.018	–	91% (20 days)	Baek et al., 2020
Graphene	Glucose	1,000–1 × 10^4^	64	0.91%	96% (30 days)	Maity et al., 2019
Graphene	Glucose	250–8,000	–	4.50%	95% (15 days)	Wang et al., 2019
Graphene	3-HB	10–400	1	4.50%	20 days	Martinez-Garcia et al., 2017
Graphene	Vitamin C	40–4,100	0.234	–	94% (50 days)	Das et al., 2018
CQDs	UA	0.21–13.39	1.3	–	–	Algarra et al., 2018
CQDs	AA	10–3,000, 4,000–12,000	10	3.58%	–	Wei et al., 2019
CQDs	Glucose	50–2,850	17	5.20%	–	Buk et al., 2019
C60	Glucose	10–3,000, 3,000–1.1 × 10^4^	4.3	1.16%	93% (30 days)	Shahhoseini et al., 2019
C60	Vitamin D-3	1.25–475	0.0025	2.27%	94.25% (35 days)	Anusha et al., 2020
Carbon nitride	Glucose	50–2,000	5	2.50%	90% (1 month)	Tian et al., 2019
Carbon nitride	Glucose	0–100	0.29	–	–	Xian et al., 2019

### Carbon Nanotubes

Carbon nanotubes (CNTs) are one of the most thoroughly studied materials with sp^2^ hybridized carbon bonds (Yang et al., 2019), which usually include multi-walled carbon nanotubes (MWCNTs), and single-walled carbon nanotube (SWCNT) (Quintero-Jaime et al., 2019). Their fascinating structures have attracted attention of numerous researchers. CNTs are seamless nanoscale tubes made of a single or multilayer graphite coiled at a certain helical angle around a central axis (De Volder et al., 2013; Noyce et al., 2019). Because of its wide range of thermal, structural, and electronic properties, they have been applied to catalyst and capacitor material support (Gong et al., 2013; Yang et al., 2017). And owing to the unique stability of the side wall and tubular structure of carbon nanotubes, they also could be used as an ideal template or carrier for other nano materials, especially nanowires (El-Nashar et al., 2012).

In 2017, Rajabi and Noroozifar (2017) used a new synthesis method to synthesize the peg-methoxyaniline nanostructure, and then successfully constructed a MWCNT of the star. The electrochemical biosensor constructed by this method had the advantages of sensitivity, simplicity, and low cost, which can simultaneously detect UA and folic acid in the real samples. In 2018, Abellan-Llobregat et al. (2018) developed a novel portable electrochemical sensor to detect AA and UA simultaneously. The sensitivity and reproducibility were tested and the results were favorable. This method had been triumphantly applied to the real samples. In the next year, Lv et al. (2019) used an electrochemical sensor constructed by porous g-C_3_N_4_ (PCN) and MWCNTs to detect UA. Owing to the PCN obtained by this method had the advantages of large specific surface area, excellent dispersion, favorable for electrocatalysis, and outstanding biocompatibility, the conductive properties of PCN were improved by using MWCNTs as conductive substrates. Zhang S. et al. (2019) prepared a novel electrochemical sensor that can detect AA and UA simultaneously. In the presence of MWCNTs, nanocomposites were prepared by *in-situ* oxidation polymerization of N-(ferrocenyl formyl) pyrrole. This biosensor has the potential to be a helpful tool for simultaneously detecting AA and UA. In addition to using metal ions, uricase can also be used as a carrier to detect UA. For example, Fukuda et al. (2020) used CNTs and uricase to form an electric-current thin film biosensor. The data detected from actual physiological samples were quantified and plotted, which were consistent with the data collected from conventional analytical methods (enzyme colorimetry kit). Manjunatha et al. (2018) constructed electrochemical biosensors by manufacturing a stable electroactive thin film of polymer onto the surface of carbon nanotubes paste electrode. Compared with bare electrode, the improved electrode had enhanced peak current and clear peak separation.

### Graphene

Graphene, as a novel nanomaterial, has received considerable attention since its successful separation (Meng et al., 2016; Saleh and Fadillah, 2019). Moreover, it has the advantages of high electron mobility, high strength, and flexibility, and plays a crucial role in biological sensing applications (Mackin and Palacios, 2016; Hou et al., 2017). At present, graphene is an important material for basic science (AlAqad et al., 2017; Zhao et al., 2018).

For UA detection, Pang et al. (2020) constructed an electrochemical sensor using a composite self-assembled from nitrogen-doped graphene/polyelectrolyte polydiallyldimethylammonium/gold nanoparticles. According to the experimental results, the prepared nanocomposites had obvious electrical activity, reproducibility, and sensitivity to UA oxidation which suggested that the sensor had the potential to diagnose uric acid-related diseases. In 2019, Sriram et al. (2019) modified reduced graphene oxide and superactive iron oxide nanospheres to assemble a novel nanocomposite, which was used to detect UA. According to the characterization results, the improved electrode had an eminent electrochemical reduction peak. In the same year, Verma et al. (2019) used gold nanoparticles to decorate graphene oxide nanocomposite thin films, which were covalently immobilized with uricase enzyme to detect UA sensitively and selectively. The interference in the real samples was evaluated, and the mixed samples were measured by UA. This biosensor had a favorable repeatability, indicating a promising future for gold nanocomposites as effective sensor substrates for biosensor applications. Hsine et al. (2020) designed an efficient and simple nanocomposite material, which was a combination of reduced graphene oxide and redox poly (para-phenylene) (Fc-ac-PP) modified with ferrrocenyl group CRGO/Fc-ac-PPP in a lateral position. The nanocomposites were able to detect AA and UA in a co-existing system by using the separated redox peaks. Demirkan et al. (2020) also detected AA and UA by preparing a novel electrochemical sensor, which was made by modifying palladium nanoparticles supported by polypyrrole/pd-reduced graphene oxide on glassy carbon electrode. Satisfactory results had been obtained when the sensor detected AA and UA in real urine samples in vivo, whether the concentration is high or not.

**Figure 2:**
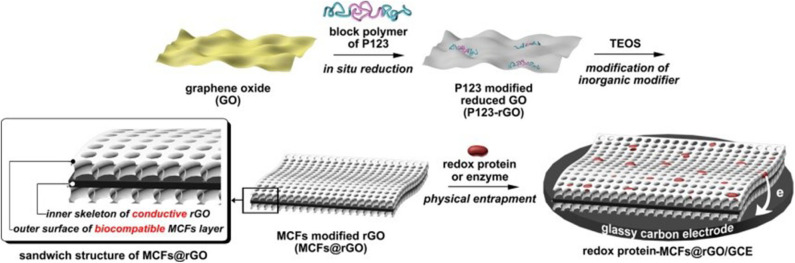
Fabrication of the MCF@rGO composite for the achievement of DET.

For the detection of vitamin C, Das et al. (2018) used the nanocomposite of iron oxide-polyvinyl alcohol to modify graphene. The SWV technique was used for detecting the biomolecule which showed that this material provided excellent results for detection of vitamin C. For the detection of NAFLD, Cai et al. (2019) prepared an environmentally friendly and unlabeled immunosensor, which was made of a composite material for the detection of leptin. This composite material was conjugated by strongly coupling between porous graphene surface plasma and anisotropic black phosphorus local surface plasma. Leptin antibodies can be firmly immobilized by the crosslinking of glutaraldehyde. Acetaminophen can be also used as a biomarker for the NAFLD, for which Kumar et al. (2019) developed an electrochemical sensor probe to detect acetaminophen. They used a crucible method to directly synthesize monodisperse iron-gold bimetallic nanoparticles, which were then impregnated with reduced graphene and coated with glassy carbon electrode. The sensor had excellent stability up to 12 weeks. Therefore, the developed sensor system had numerous outstanding characteristics, which can be widely used in clinical and pharmaceutical samples. Baek et al. (2020) constructed a biosensor by synthesizing the excellent nanocomposites to detect glucose. They used copper-nanoflower to decorate gold nanoparticles-graphene oxide nanofiber. Based on the experimental results, the improved sensor can be used to monitor glucose levels in biological fluids for real clinical care site tests. Maity et al. (2019) established a highly sensitive glucose biosensor by using an amine terminated MWCNTs/polyaniline/reduced graphene oxide/gold nanoparticles to modify screen-printed carbon electrode followed by linking glucose oxidase on the surface of electrode. Wang et al. (2019) developed a new nanomaterial by meso-cellular silicate and foam reduced graphene oxide to detect glucose, which exhibited good analytical performance for the detection of glucose. In addition, blood glucose detection in samples of human serum was successfully achieved using this sensor. The schematic diagram is shown in Figure 2.

Martinez-Garcia et al. (2017) prepared a biosensor for the detection of 3-HB by the screen-printed carbon electrode and reduced graphene oxide. An obvious result was that the improved biosensor had advantages in sensitivity compared with the bare sensor.

### Carbon Quantum Dots

Carbon quantum dots (CQDs) have been of great interest to the research community for their unique properties since they were discovered in 2004 (Algarra et al., 2018). CQDs have attracted much attention for their excellent performance such as the outstanding biocompatibility (Rombout et al., 2014; Hou et al., 2018; Zheng et al., 2018). The diameter of CQDs is <10 nm and the ratio of surface to volume is large. In addition, the surface of most CQDs contains free groups, with excellent water solubility and surface modification (Li et al., 2012; Zou et al., 2020).

Algarra et al. (2018) developed an innovative biosensor to detect UA. They used the CQDs to modify the original electrode. Fourier infrared, Fluorescence, and Raman spectra were used to characterize this material. Owing to large number of carboxyl groups on the surface of CQDs, the sensitivity of this modified glassy carbon electrode was almost 10 times that of the unmodified glassy carbon electrode. In the second year, Wei et al. (2019) established a new electrochemical sensor to quickly and accurately detect AA. CQDs were synthesized by the new method which was decorated to a glass carbon electrode by electrodeposition. Meanwhile, Buk et al. (2019) developed a new electrochemical biosensor using a hybrid nanomaterial, which was based on CQDs and gold nanoparticles. They selected glucose oxidase as a model system to validate the potential of CQDs as an immobilized substrate. According to the result, the improved biosensor had obvious advantages in the sensitivity.

### Fullerene

Fullerene, also known as C60, is a three-dimensional composite material with unique cage structure. Owing to its unique molecular configuration, its physical and chemical properties are also special (Afreen et al., 2015; Liu G.F. et al., 2018). It has a wide range of applications in nanomedicine, renewable energy industry, and electrochemical detection (Zheng et al., 2017; Xie and Zhou, 2018).

Shahhoseini et al. (2019) developed a new non-enzymatic electrocatalytic biosensor using C60 and Ni(II)-one dimensional coordination polymer. This electrochemical sensor yielded very high results that the improved biosensor had obvious advantages in the stability and sensitivity. As for vitamin D-3, Anusha et al. (2020) designed fullerene and copper-nickel bimetallic nanoparticles nanocomposite film modified glassy carbon electrode as an electrochemical sensor. The fabricated sensor employed for real samples that showed satisfactory results for the detection of vitamin D-3 in clinical and pharmaceutical samples.

### Carbon Nitride

Carbon nitride is a 2D material with special electrical properties due to its higher nitrogen content. It can be useful for various applications, particularly in energy-related fields (Heo et al., 2020).

Tian et al. (2019) synthesized 2-dimensional graphitic carbon nitride through hydrothermal method to detect glucose, and the detection limit was evaluated as 5 μM. However, Xian et al. (2019) detected glucose by developing irson-doped carbon nitride nanoparticles which were prepared from citric acid, urea, and ferric chloride through a convenient one-pot solvothermal method, a novel sensitive method for the detection of glucose with a limit of detection of 0.29 μM has been developed. The carbon nitride with mixed iron were more sensitive.

## Summary and Outlook

In summary, we summarized the latest research progress of electrochemical sensors based on carbon nanomaterials in metabolic disease markers in the last 3 years including carbon nanotubes, graphene, carbon quantum dots, fullerenes, and carbon nitrates. Metabolic diseases not only affect normal diet and life of people but also seriously threaten human health and safety. In addition to maintaining a healthy lifestyle and eating with a healthy diet, early diagnosis of the markers of metabolic disease is also essential which mainly include glucose, uric acid, leptin, and various vitamins. As a result, it is of great significance to develop prompt and sensitive early diagnosis and detection technology to control these diseases.

So far, the electrochemical immunosensor used to analyze actual samples has been widely reported, especially for the detection of glucose and ascorbic acid. Generally speaking, its minimum detection threshold is lower than that of other traditional methods. However, due to some technical and commercial reasons, electrochemical immunosensors have not become the mainstream of practical application. For example, the reproducibility and stability of many electrochemical immunosensors prepared as shown in Table 1 are not mentioned, so it is a new challenge to improve the stability and reproducibility of the electrode interface. Moreover, the miniature or portable electrochemical devices are rarely reported for the diagnosis of metabolic diseases, especially for clinical application. Therefore, the research on development of portable, low-cost nano-electrochemical sensors for the diagnosis of metabolic disease has the potential to become a major focus of the research in the near future.

## Author Contributions

CZ: conceptualization and writing—original draft preparation. XD: validation, writing—review and editing, and project administration. All authors contributed to the article and approved the submitted version.

## Conflict of Interest

The authors declare that the research was conducted in the absence of any commercial or financial relationships that could be construed as a potential conflict of interest.
